# Multi‐Target Repetitive Transcranial Magnetic Stimulation Improves Freezing of Gait in Parkinson's Disease: A Randomized Controlled Trial

**DOI:** 10.1111/cns.70582

**Published:** 2025-08-22

**Authors:** Zixuan Zhang, Danyang Liu, Wenjing Song, Jinyu Li, Xi Wang, Peixiao Yin, Yuning Liu, Min Xu, Fujia Li, Yumeng Li, Guiyun Cui, Wei Zhang

**Affiliations:** ^1^ Department of Neurology The Affiliated Hospital of Xuzhou Medical University Xuzhou Jiangsu China; ^2^ Department of Neurology The First Clinical College, Xuzhou Medical University Xuzhou Jiangsu China

**Keywords:** freezing of gait, motor cortex, Parkinson's disease, repetitive transcranial magnetic stimulation, supplementary motor area

## Abstract

**Objective:**

In this randomized, double‐blind, sham‐controlled trial, we investigated the efficacy of multi‐target 10‐Hz rTMS targeting both M1 and SMA in alleviating freezing of gait (FOG) in Parkinson's disease (PD) patients.

**Methods:**

Eighty‐four PD‐FOG patients were randomly assigned (1:1:1:1) to four groups: M1 and SMA group, M1 group, SMA group, and sham group. rTMS was administered once daily for 10 consecutive days. Assessments were conducted at baseline (T0), after the final session (T1), and 30 days posttreatment (T2), using the FOG‐Q, UPDRS‐III, Timed Up and Go (TUG) Test, Standing‐Start 180° Turn (SS‐180) Test, and measures of emotion and cognition.

**Results:**

Application of 10‐Hz rTMS to bilateral M1 or SMA significantly reduced FOG severity, improved motor function, and alleviated emotional disturbances in PD patients, with effects lasting at least 1 month. Compared to single‐target stimulation, multi‐target stimulation of M1 and SMA high‐frequency rTMS showed more pronounced therapeutic effects across these outcomes. However, no significant cognitive improvements were observed in either the real or sham stimulation groups.

**Conclusions:**

The results of this study indicate that bilateral M1 and SMA 10‐Hz rTMS is a promising therapeutic approach, providing new possibilities for clinical treatment.

## Graphical abstract

10‐Hz rTMS targeting bilateral M1 or SMA alleviates FOG, improves motor function, and enhances emotion in PD patients, with effects lasting over a month. Multi‐target stimulation surpasses single‐target protocol, highlighting its therapeutic potential. No severe adverse effects were reported, reinforcing rTMS as a safe treatment.

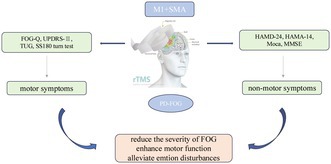

## 1 Introduction

Parkinson's disease (PD) is a prevalent neurodegenerative disorder characterized by motor symptoms such as bradykinesia, resting tremor, rigidity, and disturbances in posture and gait, alongside a range of non‐motor symptoms [1, 2, 3]. Freezing of gait (FOG) is defined as a brief, sudden cessation or marked reduction in stride during walking, typically presenting as motor block, difficulty, or hesitation in initiating movement. In severe cases, it leads to frequent falls, increasing disability, and significantly diminishing patients' quality of life [4, 5].

Currently, the treatment of FOG faces multiple challenges [6], with existing approaches including pharmacological interventions, deep brain stimulation (DBS), and gait rehabilitation training. Due to the heterogeneity of its underlying pathophysiology, the complexity of the neural regulatory network, and individual patient variability, none of these methods offer an optimal solution for treating FOG [7, 8, 9, 10]. In recent years, repetitive transcranial magnetic stimulation (rTMS), a noninvasive brain stimulation (NIBS) technique, has garnered increasing attention from researchers [11]. By delivering repeated and systematic stimulation to specific cortical regions, rTMS modulates local cortical electrical activity and, through the regulation of cortical–cortical and cortical–subcortical networks, influences the function of distant brain areas [12, 13].

During rTMS intervention, the coil generates a pulsatile magnetic field that penetrates the scalp and skull [14], modulating cortical excitability depending on the stimulation frequency: high‐frequency stimulation (≥ 5 Hz) is generally excitatory, whereas low‐frequency stimulation (≤ 1 Hz) is inhibitory [15, 16]. Based on this principle, multiple studies targeting PD‐FOG have employed high‐frequency rTMS (HF‐rTMS), for example, 5 or 10 Hz, to enhance excitability in motor‐related cortical areas, often demonstrating gait improvements superior to sham stimulation [17, 18]. Commonly targeted regions include the primary motor cortex (M1) [12], supplementary motor area (SMA) [19, 20], and dorsolateral prefrontal cortex (DLPFC) [21, 22]. HF‐rTMS applied to bilateral M1 has been shown to improve step count, gait timing, and Freezing of Gait Questionnaire (FOG‐Q) scores, with effects lasting up to 1 week posttreatment [22]. Another small‐scale study divided PD‐FOG patients into M1 and SMA groups, each receiving 25‐Hz rTMS stimulation for two consecutive days. The results revealed a significantly greater reduction in freezing episodes during the TUG test in the SMA group compared to the M1 group, suggesting that SMA may be more effective than M1 [23]. Yokoe et al. [24] were the first to explore the effects of targeting M1, SMA, and DLPFC on motor function improvement. Their findings confirmed that 10‐Hz rTMS applied to M1 or SMA significantly improved motor symptoms in PD patients, whereas the UPDRS‐III scores following DLPFC stimulation were similar to those of the sham stimulation group, indicating that DLPFC stimulation has limited efficacy in improving motor symptoms in PD. Research indicates that the M1 is closely involved in the control of lower limb movements. HF‐rTMS applied to M1 can partially compensate for the reduced output of the basal ganglia‐thalamocortical pathway to the frontal motor cortical areas, leading to sustained enhancement of cortical excitability and subsequent motor improvement. The SMA transmits information regarding the postural muscle tone required for specific gait patterns via the cortico‐reticular and reticulospinal tracts, thereby modulating gait initiation. Notably, the SMA and M1 exhibit functional coupling in motor control, collaboratively facilitating the output of the corticospinal tract and generating spontaneous motor commands [25]. While brain regions such as the prefrontal cortex, basal ganglia, and cingulate cortex are also implicated in motor function, they indirectly influence motor control through extensive neural networks. In contrast, M1 and SMA have direct roles in gait regulation, making them optimal targets for rTMS interventions.

Our preliminary studies demonstrated that HF‐rTMS targeting bilateral M1 improves gait freezing, walking ability, motor function, and emotional disturbances in PD patients [26]. However, most research has focused on single‐target rTMS for PD‐FOG. Given that FOG involves dysfunction across multiple brain regions and neural networks, particularly those regulating motor control and gait planning, we hypothesize that multi‐target rTMS targeting both M1 and SMA offers superior efficacy over single‐site stimulation in alleviating FOG severity, enhancing motor function, and improving emotional disturbances. This study aims to investigate the therapeutic effects of the multi‐target stimulation of M1 and SMA in PD patients with FOG.

## 2 Methods

### 2.1 Study Participants

Eighty‐six PD‐FOG patients were consecutively recruited from the Affiliated Hospital of Xuzhou Medical University. All participants were diagnosed with idiopathic PD according to the diagnostic criteria set forth by the International Parkinson and Movement Disorder Society (MDS). The inclusion criteria were as follows: (1) age between 45 and 80 years, (2) diagnosis of FOG meeting at least one of the following criteria [27, 28]: self‐reported sensation of ‘feet stuck to the ground’ during initiation, turning, or walking, as assessed by item 3 of the FOG‐Q [29]; FOG episodes were confirmed by presenting typical video recordings of FOG events to both the patient and caregiver; FOG was triggered by bidirectional 360°rapid turns, dual‐tasking, and small‐step fast walking, (3) Hoehn‐Yahr stage 2–4 [30], (4) Mini‐Mental State Examination (MMSE) score ≥ 24 [31], (5) stable medication regimen for at least 2 weeks prior to participation, with no changes during the study period, (6) ability to cooperate with rTMS treatment and symptom assessment.

**FIGURE 1:**
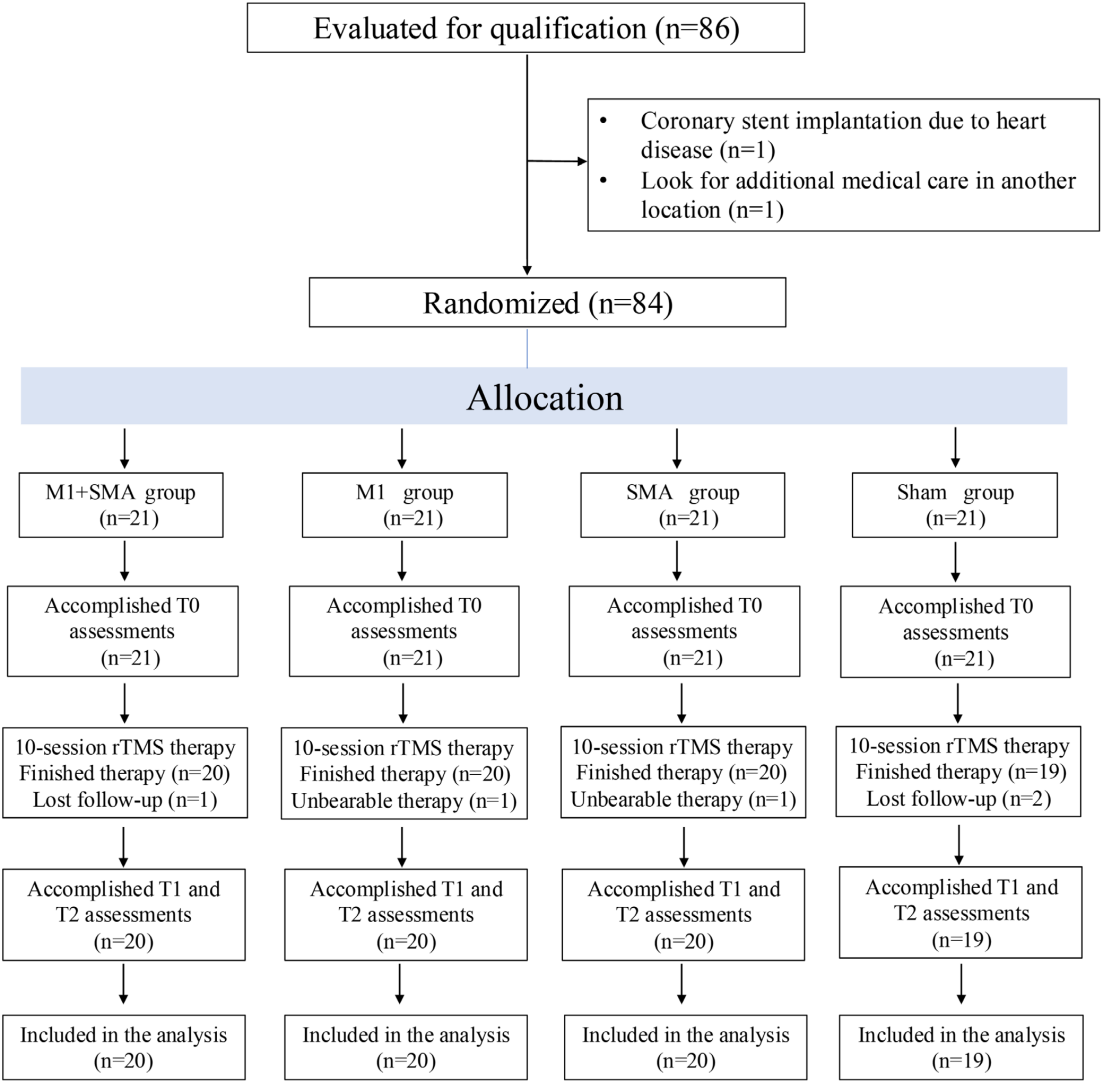
Study flow diagram. T0, prior to the first rTMS session; T1, after the 10th treatment session; T2, 30 days after T1. M1 + SMA group, M1 + SMA combined target rTMS group; M1 group, M1 single‐target rTMS group; SMA group, SMA single‐target rTMS group; Sham group, Sham rTMS group.

The exclusion criteria were as follows: (1) contraindications to TMS treatment, (2) based on a comprehensive evaluation of medical history, clinical presentation, and neuroimaging findings, patients were considered to be at risk for Parkinson‐plus syndromes, secondary parkinsonism, vascular dementia, normal pressure hydrocephalus, or other major neurological disorders such as intracranial mass lesions or stroke, (3) previous TMS or transcranial direct current stimulation (tDCS) treatment, (4) prominent rest tremor that would interfere with maintaining head immobility during rTMS treatment. Prior to randomization, one participant was excluded due to the implantation of a coronary artery stent, and one withdrew for personal reasons. Thus, a total of 84 PD‐FOG patients ultimately participated in the clinical trial (Figure 1).

This study was approved by the Ethics Committee of the Affiliated Hospital of Xuzhou Medical University (No. XYFY2024‐KL126‐01). Prior to participation, all subjects provided written informed consent in accordance with the Declaration of Helsinki. The study has been registered on the Clinical Trial Registry (https://www.chictr.org.cn) under the registration number ChiCTR2400091434.

### 2.2 Study Design

This randomized, double‐blind, placebo‐controlled trial assigned participants into four groups using R software: M1 + SMA combined target rTMS group (*n* = 21), M1 single‐target rTMS group (*n* = 21), SMA single‐target rTMS group (*n* = 21), and Sham group (*n* = 21). Each group underwent 10 intervention sessions over 10 days. Group allocation remained blinded to both participants and researchers, with randomization sequences disclosed only to the clinicians administering rTMS. During the blind validation process, participants were periodically asked to guess their assigned group, and statistical analysis was conducted to assess the effectiveness of blinding. All participants were receiving standard anti‐PD pharmacotherapy, with their medication regimen remaining unchanged throughout the study, ensuring that rTMS treatment effects were not confounded by adjustments in drug dosage. Furthermore, all rTMS sessions were scheduled at relatively fixed time windows when patients were in the medication “ON” state. Treatment evaluations were also mostly conducted around 10 a.m. to minimize the potential confounding effects of medication fluctuations and timing variability on clinical outcomes.

### 2.3 rTMS and Sham Protocols

Participants received real rTMS or sham rTMS for 10 consecutive days, administered by trained professionals. rTMS was delivered using the Magstim Rapid2 transcranial magnetic stimulation system, equipped with a 70‐mm figure‐eight coil. Stimulation targeted the M1 and SMA, administered in the sequence of M1 then SMA, with the more affected hemisphere targeted first, followed by the contralateral hemisphere. The center of the figure‐eight coil was positioned over the surface area corresponding to the lower limb representation in the bilateral M1. Following M1 stimulation, SMA stimulation was localized 3 cm anterior to Cz along the sagittal midline [32]. Each participant received 20 min of rTMS treatment targeting a single site on one hemisphere, consisting of 1200 pulses of 10‐Hz rTMS at 100% resting motor threshold (RMT) intensity. Each pulse lasted 2 s, with an 18‐s inter‐pulse interval. RMT was defined as the minimum stimulation intensity required to elicit a motor‐evoked potential (MEP) of at least 50 μV in the first dorsal interosseous (FDI) muscle, successfully induced in at least five out of 10 consecutive trials, when the muscle was in a relaxed state.

The Sham rTMS group received identical parameters (including frequency, intensity, and pulse sequence) as the real stimulation group, but the stimulation coil was rotated 90° to align its magnetic field vector parallel to the cortical surface, thereby reducing the magnetic field strength at the target area to near zero and eliminating the neuromodulatory effect [33]. This configuration maintained auditory and scalp sensations comparable to those in the real stimulation group. In the single‐target stimulation groups, the non‐target site was subjected to Sham rTMS.

### 2.4 Clinical Assessments

**FIGURE 2:**
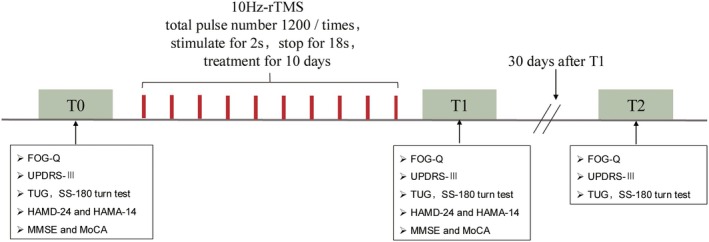
Flow chart of the experimental design. T0, prior to the first rTMS session; T1, after the 10th treatment session; T2, 30 days after T1.

Clinical symptoms were evaluated at three time points: prior to the first rTMS session (T0); after the tenth treatment session (T1); and 30 days after T1 (T2) (Figure 2).

**FIGURE 3:**
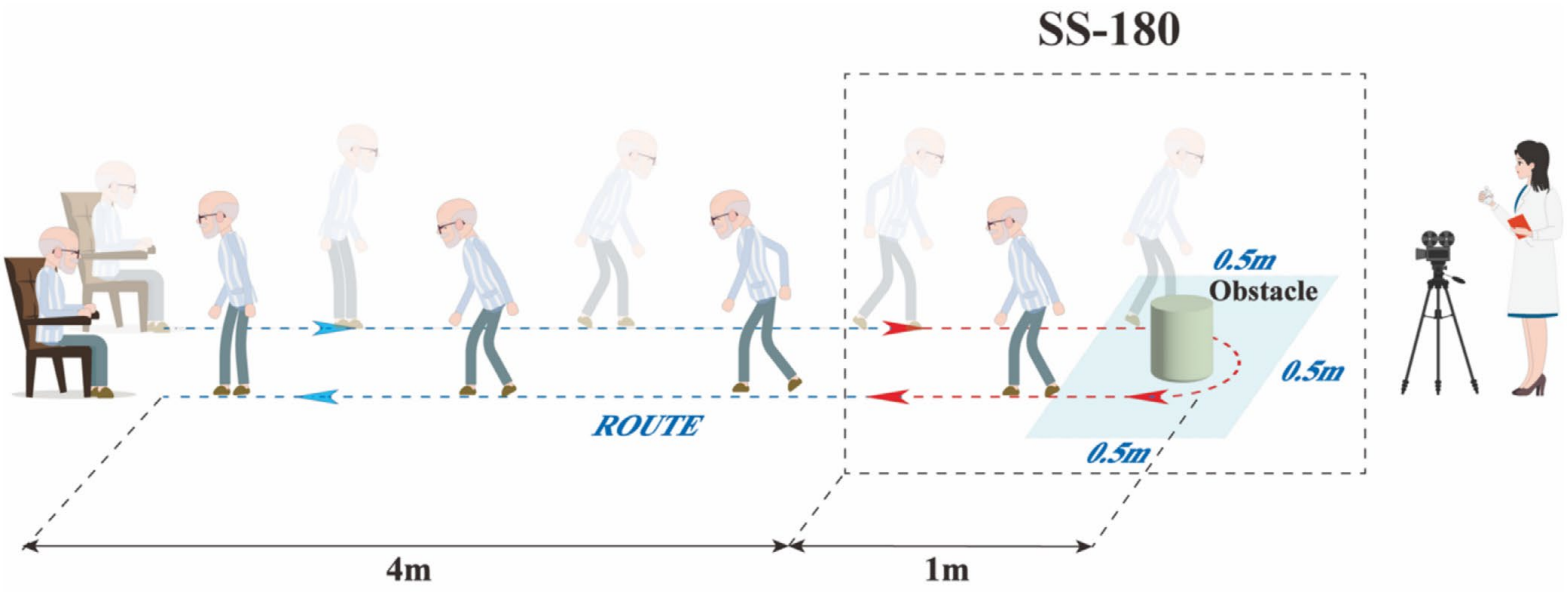
Schematic diagram of TUG and SS‐180 test methods. TUG and SS‐180 Test Procedures: A cylindrical obstacle was placed 5 m in front of the participant's chair within a marked 0.5 m × 0.5 m area. At the “start” command, participants stood up, walked 4 m, turned around the obstacle within the designated area, returned, and sat down. Three parameters were recorded: (1)Total TUG Time (2)SS‐180 Turn Time (3)SS‐180 Turn Steps.

This study utilized the internationally recognized FOG‐Q as the primary efficacy outcome, while the UPDRS‐III score served as a secondary measure to assess the impact of rTMS on global motor function. Since the FOG‐Q is a subjective measure for evaluating FOG severity, and turning movements, particularly 180°and 360°turns, are commonly used to elicit FOG in objective assessments [33], we included the Timed Up and Go (TUG) test and the Standing‐Start 180°Turn (SS‐180) test as secondary efficacy measures. In the TUG test, participants rise from a seated position, walk 5 m, turn around an obstacle, and return to the chair, with total time recorded. In the SS‐180°turn test, participants start from a standing position 1 m from the obstacle, with both turn time (SS‐180 turn time) and the number of steps taken (SS‐180 turn steps) measured. Each side is tested twice, and the average value is used for analysis (Figure 3).

Among PD's non‐motor symptoms [34], depression, anxiety, and cognitive impairment are key risk factors for FOG, exerting a bidirectional influence on gait freezing [35, 36, 37]. Therefore, this study evaluates rTMS effects on cognitive function using the mini‐mental state examination (MMSE) and the Montreal cognitive assessment (MoCA), while the 24‐item Hamilton depression scale (HAMD‐24) and the 14‐item Hamilton anxiety scale (HAMA‐14) assess its efficacy in alleviating depression and anxiety.

### 2.5 Statistics Analysis

The required sample size was calculated using G*Power software (Version 3.1.9.2) with the following parameters: effect size f = 0.25, α = 0.01, power = 0.8, number of groups = 4, and number of measurements = 3. The calculation determined that a total sample size of 56 participants was required. To ensure robust statistical power, a total of 84 participants were ultimately enrolled based on stringent inclusion and exclusion criteria.

Data analysis was performed using SPSS 26.0 (IBM, Chicago, IL, USA). Continuous variables were reported as means ± standard deviations (SD) or medians with interquartile ranges (IQR), depending on whether the data were normally distributed, while categorical variables were presented as frequencies and percentages. Group comparisons were conducted using one‐way ANOVA or the Kruskal–Wallis test. Two‐way repeated measures ANOVA assessed the main and interaction effects of time points (within‐factor: T0, T1, T2) and groups (between‐factor: M1 + SMA, M1, SMA, Sham). Significant main effects were further analyzed using Bonferroni‐corrected post hoc tests, while interaction effects were examined through simple effect analysis. Spearman correlation was used to explore associations between changes in emotion and cognitive scores (△HAMD‐24, △HAMA‐14, △MoCA, △MMSE) and changes in FOG‐Q (△FOG‐Q) within the real stimulation group. The level of statistical significance was set at *p* < 0.05.

## 3 Results

### 3.1 Baseline Characteristics of All Subjects

Table 1 summarizes the demographic and baseline clinical characteristics of the M1 + SMA, M1, SMA, and Sham groups. No significant differences were found across groups in sex, age, disease duration, H&Y stage, LEDD, FOG‐Q, UPDRS‐III, HAMD‐24, HAMA‐14, MMSE, or MoCA scores.

**TABLE 1:** Demographic and clinical characteristics of all subjects.

Variables	rTMS group (*n* = 60)			Sham group (*n* = 19)	*p*
	M1 + SMA (*n* = 20)	M1 (*n* = 20)	SMA (*n* = 20)		
Gender (female/male)	10/10	7/13	9/11	11/8	0.624
Age (years)	69.20 ± 5.78	69.05 ± 6.75	67.40 ± 7.22	71.40 ± 6.02	0.285
Disease duration (years)	7.15 ± 3.25	7.35 ± 2.88	7.05 ± 2.41	7.75 ± 2.57	0.477
H‐Y stage	2.5 (2.125, 3.0)	3 (2.5, 3.0)	2.5 (2.0,3.0)	3 (2.5, 3.0)	0.076
LEDD (mg/d)	721.25 ± 274.11	712.50 ± 233.72	770.46 ± 258.47	740.35 ± 185.36	0.876
FOG‐Q	14.20 ± 2.40	14.25 ± 3.39	14.45 ± 2.26	14.40 ± 2.54	0.989
UPDRS‐III (ON medication)	41.30 ± 7.94	40.90 ± 7.40	40.85 ± 6.97	42.20 ± 7.71	0.937
HAMD‐24	16.85 ± 2.54	16.85 ± 3.25	16.80 ± 2.78	16.75 ± 2.55	0.999
HAMA‐14	10.5 (8.25, 12)	10.5 (10, 12.75)	10 (9,13)	9 (8, 11)	0.076
MMSE	27 (25.25, 29)	26.5 (25, 28)	28 (25.25,29)	26 (25, 27)	0.087
MoCA	23.55 ± 2.21	22.35 ± 3.69	24.25 ± 2.02	22.00 ± 3.46	0.063

Abbreviations: FOG‐Q, freezing of gait questionnaire; HAMA‐14, Hamilton anxiety rating scale‐14 item; HAMD‐24, Hamilton depression rating scale‐24 item; H‐Y stage, Hoehn and Yahr stage; LEDD, levodopa‐equivalent daily dose; MDS‐UPDRS III, movement disorder society unified Parkinson's disease rating scale part III; MMSE, mini‐mental state examination; MoCA, Montreal cognitive assessment; rTMS, repetitive transcranial magnetic stimulation.

### 3.2 Adverse Effects Assessments

No serious adverse events occurred during rTMS treatment. However, five participants reported mild discomfort: two experienced headaches, two had dizziness with nausea, and one developed tinnitus. We immediately discontinued treatment and assessed the participants' vital signs. While symptoms resolved spontaneously within hours, all five withdrew from the study.

### 3.3 Alterations in FOG Evaluations: FOG‐Q

**FIGURE 4:**
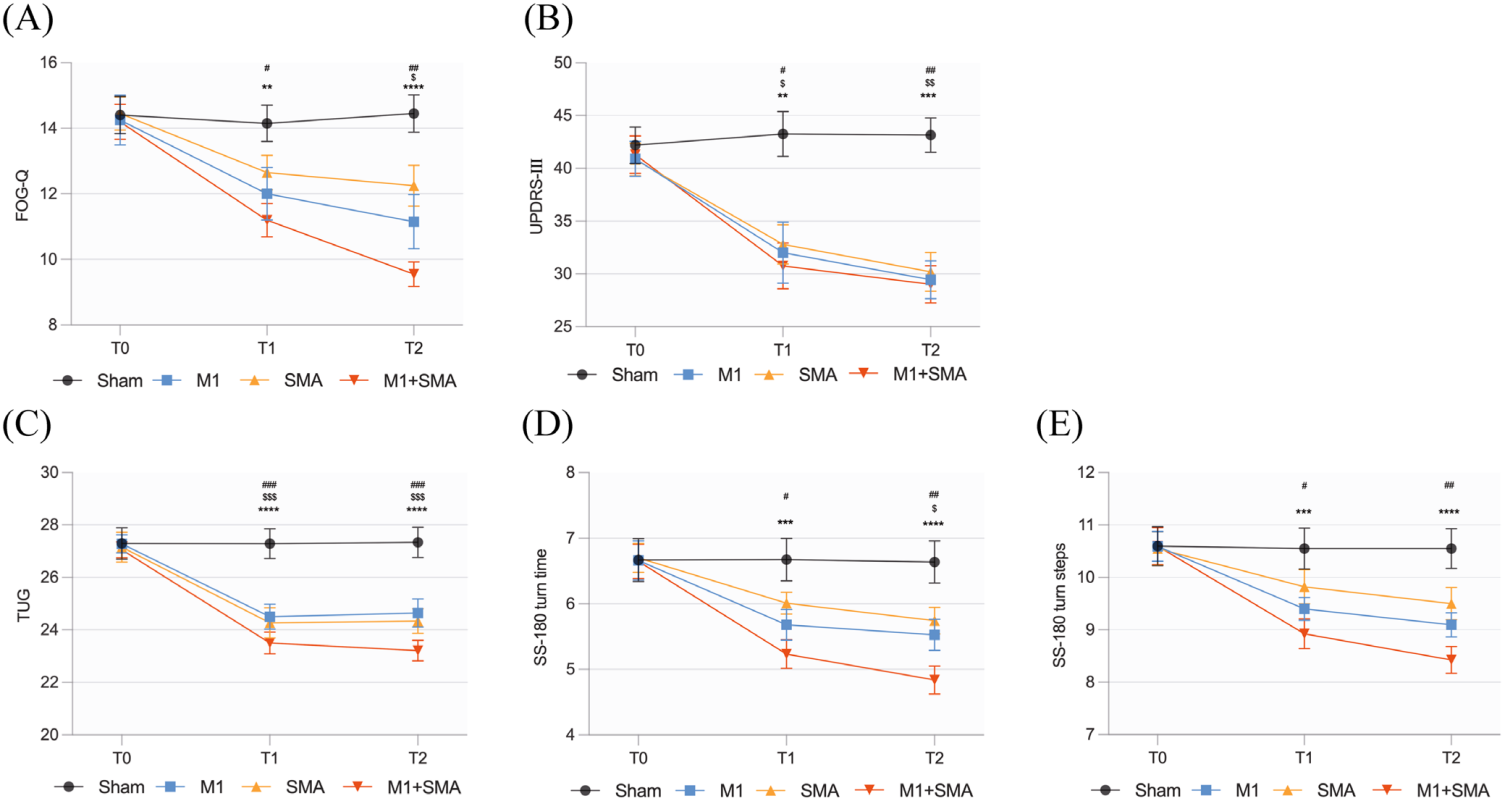
Clinical improvements following rTMS therapy in four groups. (A) FOG‐Q, (B) UPDRS‐III, (C) TUG, (D) SS‐180 turn time, (E) SS‐180 turn steps. *, M1 + SMA group; #, M1 group; $, SMA group.*/#/$ *p* < 0.05; **/##/$$ *p* < 0.01; ***/###/$$$ *p* < 0.001; **** *p* < 0.0001.

TIME (F_TIME_ = 180.206, *p* < 0.001), GROUP (F_GROUP_ = 3.696, *p* = 0.015), and their interaction (F_TIME×GROUP_ = 26.785, *p* < 0.001) had a significant effect on FOG‐Q. Compared to T0, the M1 + SMA and M1 groups showed significant improvement at T1 and T2 (*p* < 0.05). The SMA group exhibited a downward trend at T1 without statistical significance, but showed significant improvement at T2 (*p* < 0.05) (Figure 4). Notably, symptom relief was greater in the M1 + SMA group (ΔFOG‐Q_M1+SMA_ at T1: −3.00 ± 1.17; ΔFOG‐Q_M1+SMA_ at T2: −4.65 ± 1.63) than in the single‐target groups (ΔFOG‐Q_M1_ at T1: −2.25 ± 1.07; ΔFOG‐Q_SMA_ at T1: −1.80 ± 1.64; ΔFOG‐Q_M1_ at T2: −3.10 ± 1.65; ΔFOG‐Q_SMA_ at T2: −2.20 ± 1.47) (Table 2).

**TABLE 2:** Clinical efficiency of the rTMS and sham protocol.

	M1 + SMA group	M1 group	SMA group	Sham group		DF	F	*p*
**FOG‐Q**								
T0	14.20 ± 2.40	14.25 ± 3.39	14.45 ± 2.26	14.40 ± 2.54	Group	3.000	3.696	0.015
T1	11.2 ± 2.28	12.00 ± 3.60	12.65 ± 2.37	14.15 ± 2.48	Time	1.761	180.206	0.000
T2	9.55 ± 1.67	11.15 ± 3.70	12.25 ± 2.77	14.45 ± 2.52	Group × Time	5.284	26.785	0.000
**UPDRS‐III**								
T0	41.30 ± 7.94	40.90 ± 7.40	40.85 ± 6.97	42.20 ± 7.71	Group	3.000	8.296	0.000
T1	30.75 ± 7.95	30.95 ± 8.02	32.05 ± 8.41	43.25 ± 9.57	Time	1.341	82.614	0.000
T2	29.00 ± 7.97	29.45 ± 8.04	30.20 ± 8.24	43.15 ± 7.30	Group × Time	4.024	11.803	0.000
TUG								
T0	27.05 ± 1.35	27.28 ± 1.51	27.15 ± 2.53	27.29 ± 2.70	Group	3.000	6.556	0.001
T1	23.50 ± 1.88	24.51 ± 2.12	24.27 ± 2.57	27.29 ± 2.54	Time	1.730	120.918	0.000
T2	23.22 ± 1.75	24.65 ± 2.39	24.34 ± 2.10	27.34 ± 2.58	Group × Time	5.191	14.865	0.000
**SS‐180 turn time**								
T0	6.65 ± 1.17	6.66 ± 1.33	6.70 ± 0.98	6.67 ± 1.46	Group	3.000	3.401	0.022
T1	5.24 ± 0.99	5.68 ± 1.05	6.01 ± 0.75	6.68 ± 1.45	Time	1.275	117.072	0.000
T2	4.84 ± 0.96	5.53 ± 1.06	5.75 ± 0.91	6.64 ± 1.43	Group × Time	3.826	16.775	0.000
**SS‐180 turn steps**								
T0	10.60 ± 1.59	10.60 ± 1.26	10.55 ± 1.45	10.60 ± 1.67	Group	3.000	3.054	0.033
T1	8.93 ± 1.28	9.40 ± 0.98	9.83 ± 1.46	10.55 ± 1.76	Time	1.484	101.503	0.000
T2	8.43 ± 1.16	9.10 ± 1.02	9.50 ± 1.40	10.55 ± 1.71	Group × Time	4.453	14.332	0.000
**HAMD‐24**								
T0	16.85 ± 2.54	16.85 ± 3.25	16.80 ± 2.78	16.75 ± 2.55v	Group	3.000	7.455	0.000
T1	12.60 ± 2.35	13.85 ± 2.70	14.35 ± 2.11	16.95 ± 2.98	Time	1.812	125.125	0.000
T2	10.95 ± 2.58	12.35 ± 1.69	14.45 ± 2.39	16.85 ± 2.83	Group × Time	5.437	21.570	0.000
**HAMA‐14**								
T0	9.55 ± 1.70	10.80 ± 2.50	10.50 ± 2.31	11.10 ± 1.65	Group	3.000	14.622	0.000
T1	6.00 ± 2.29	8.55 ± 2.67	8.85 ± 1.98	10.95 ± 1.47	Time	1.817	196.320	0.000
T2	5.40 ± 1.98	7.15 ± 2.23	8.25 ± 2.02	11.00 ± 1.38	Group × Time	5.450	28.947	0.000

*Note:* Values were presented as mean ± SD.

Abbreviations: FOG‐Q, freezing of gait questionnaire; HAMA‐14, Hamilton anxiety rating scale‐14 item; HAMD‐24, Hamilton depression rating scale‐24 item; rTMS, repetitive transcranial magnetic stimulation; SS‐180, standing‐start 180°turn test; TUG, timed up and go test; UPDRS‐III, unified Parkinson's disease rating scale part III.

### 3.4 Alterations in Motor Function Evaluations: UPDRS‐III

Repeated ANOVA revealed significant main effects of TIME (F_TIME_ = 82.614, *p* < 0.001), GROUP (F_GROUP_ = 8.296, *p* < 0.001), and TIME × GROUP interactions (F_TIME×GROUP_ = 11.803, *p* < 0.001) on the UPDRS‐III scores. Consistent with the FOG‐Q results, the M1 + SMA group (*p* < 0.01), M1 group (*p* < 0.05), and SMA group (*p* < 0.05) all showed significant improvements at T1 and T2 compared to T0 (Figure 4). Compared to the Sham group (ΔUPDRS‐III_Sham_ at T1: 1.05 ± 5.82; ΔUPDRS‐III_Sham_ at T2: 0.95 ± 3.12), the M1 + SMA (ΔUPDRS‐III_M1+SMA_ at T1: −10.55 ± 6.95; ΔUPDRS‐III_M1+SMA_ at T2: −12.30 ± 7.89), M1 (ΔUPDRS‐III_M1_ at T1: −9.95 ± 8.20; ΔUPDRS‐III_M1_ at T2: −11.45 ± 8.74), and SMA groups (ΔUPDRS‐III_SMA_ at T1: −8.80 ± 7.56; ΔUPDRS‐III_SMA_ at T2: −10.65 ± 8.28) all showed significant motor function improvements, with the M1 + SMA group exhibiting the most pronounced effect (Table 2).

### 3.5 Alterations in Objective Gait Characteristics: TUG, SS‐180 Turn Test

Our results showed significant main effects of TIME and GROUP, as well as TIME × GROUP interactions, on TUG (F_TIME_ = 120.918, *p* < 0.001; F_GROUP_ = 6.556, *p* = 0.001; F_TIME×GROUP_ = 14.865, *p* < 0.001), SS‐180 turn time (F_TIME_ = 117.072, *p* < 0.001; F_GROUP_ = 3.401, *p* = 0.022; F_TIME×GROUP_ = 16.775, *p* < 0.001), and SS‐180 turn steps (F_TIME_ = 101.503, *p* < 0.001; F_GROUP_ = 3.054, *p* = 0.033; F_TIME×GROUP_ = 14.332, *p* < 0.001). The M1 + SMA group showed the most pronounced improvement across all metrics at T1 and T2 (*p* < 0.001). The M1 group exhibited significant improvement in TUG (*p* < 0.001) and reductions in SS‐180 turn time and steps (*p* < 0.05). The SMA group improved in TUG (*p* < 0.01), while SS‐180 turn time and steps showed a downward trend without statistical significance. No significant changes were observed in the Sham group (Figure 4).

### 3.6 Alterations in Emotion and Cognition

**FIGURE 5:**
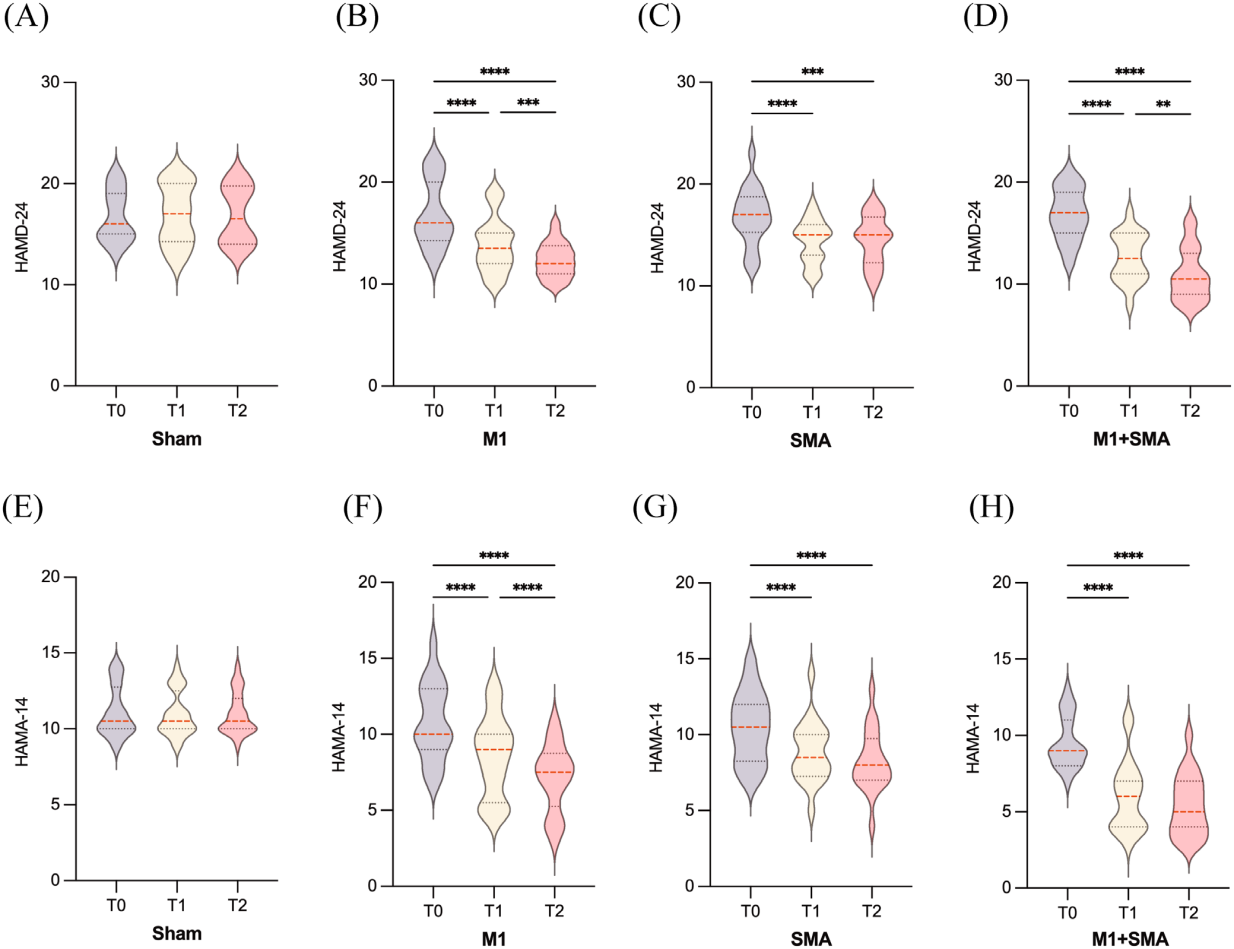
Comparison of emotion scores among M1 + SMA, M1, SMA, and Sham groups. * *p* < 0.05; ** *p* < 0.01; *** *p* < 0.001; **** *p* < 0.0001.

Repeated ANOVA disclosed significant effects of TIME and GROUP and TIME × GROUP interactions on HAMD‐24 (F_TIME_ = 125.125, *p* < 0.001; F_GROUP_ = 7.455, *p* < 0.001; F_TIME×GROUP_ = 21.57, *p* < 0.001) and HAMA‐14 (F_TIME_ = 196.320, *p* < 0.001; F_GROUP_ = 14.622, *p* < 0.001; F_TIME×GROUP_ = 28.947, *p* < 0.001). The M1 + SMA (*p* < 0.01), M1 (*p* < 0.001), and SMA groups (*p* < 0.001) showed significant reductions in both scores at T1 and T2 compared to T0. No significant difference was found between T1 and T2 in the SMA group (Figure 5).

**FIGURE 6:**
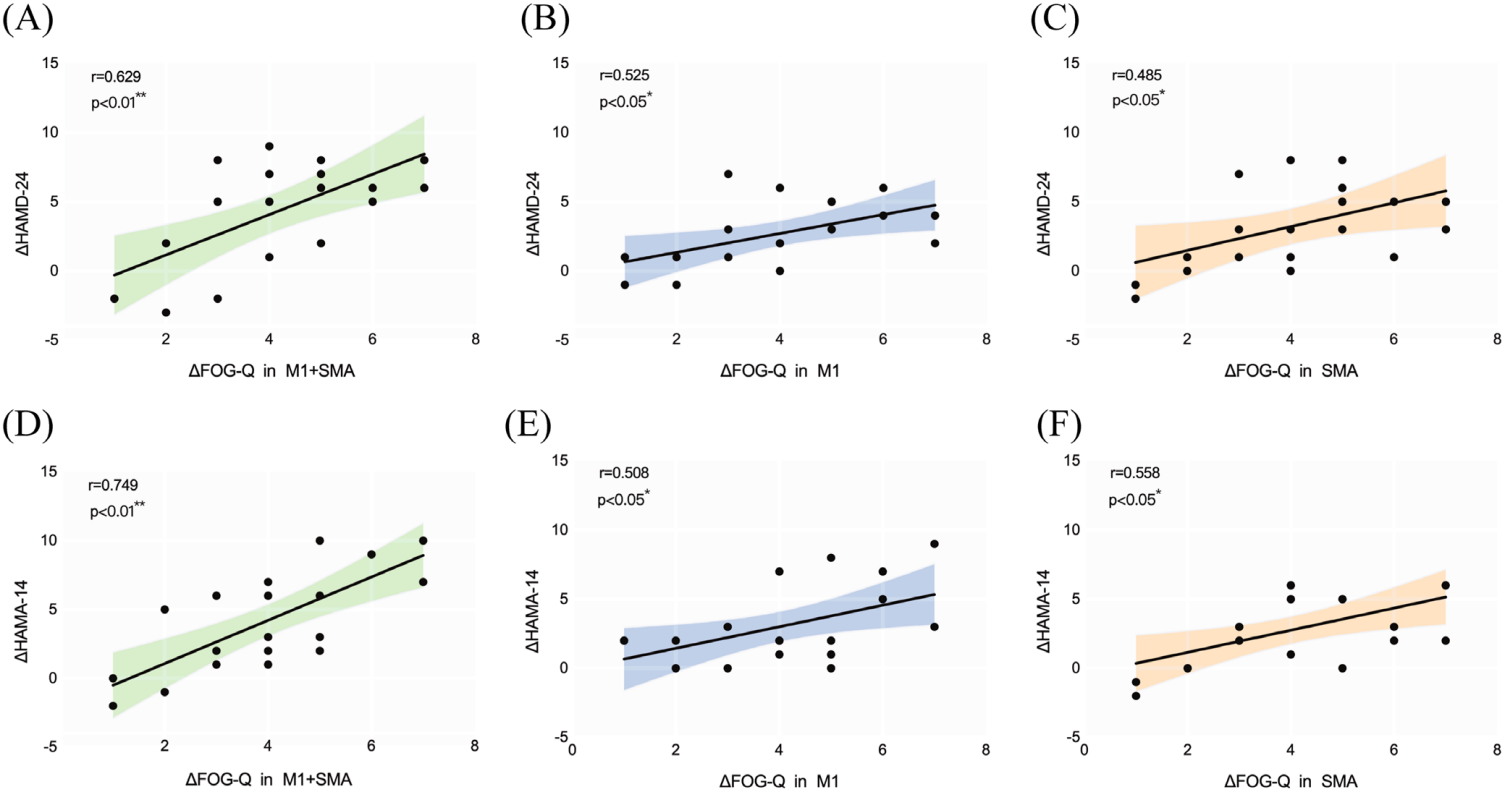
Postintervention emotional changes and their clinical correlation. (A) Correlation between HAMD‐24 and FOG‐Q changes in the M1 + SMA group; (B) Correlation between HAMD‐24 and FOG‐Q changes in the M1 group; (C) Correlation between HAMD‐24 and FOG‐Q changes in the SMA group. (D) Correlation between HAMA‐14 and FOG‐Q changes in the M1 + SMA group; (E) Correlation between HAMA‐14 and FOG‐Q changes in the M1 group; (F) Correlation between HAMA‐14 and FOG‐Q changes in the SMA group. The *p* and *r* are indicated, and *p* < 0.05 was considered significant.

Spearman correlation analysis showed a significant positive correlation between gait improvement (ΔFOG‐Q) and emotion enhancement (ΔHAMD‐24, ΔHAMA‐14) at T1 in the M1 + SMA (r_HAMD‐24_ = 0.629, *p* < 0.01; r_HAMA‐14_ = 0.749, *p* < 0.01), M1 (r_HAMD‐24_ = 0.525, *p* < 0.05; r_HAMA‐14_ = 0.508, *p* < 0.05), and SMA (r_HAMD‐24_ = 0.485, *p* < 0.05; r_HAMA‐14_ = 0.558, *p* < 0.05) groups, indicating that greater gait recovery aligns with greater emotional improvement (Figure 6). No significant changes in MMSE or MoCA were observed in either the rTMS or Sham groups across time points.

## 4 Discussion

This study evaluated the efficacy of multi‐target rTMS for treating PD‐FOG. The results show that HF‐rTMS (10 Hz) applied to bilateral M1 or SMA significantly reduces the severity of FOG, enhances motor function, and alleviates anxiety and depression. Notably, compared to single‐target rTMS, multi‐target stimulation more effectively reduces FOG frequency, shortens hesitation times before walking and movement initiation, and improves emotional disorders. Moreover, it produces long‐lasting biological effects at least 1 month posttreatment. These findings highlight the superior potential of multi‐target rTMS in FOG treatment, providing a better therapeutic strategy for PD patients. However, cognitive function improvements were limited in both the real and sham stimulation groups.

This study found that the application of 10‐Hz rTMS to bilateral M1 in patients resulted in significant improvements in the severity of FOG (T1_FOG‐Q_: −2.25, T2_FOG‐Q_: −3.10), enhanced motor function (T1_UPDRS‐III_: −9.95, T2_UPDRS‐III_: −11.45), and markedly reduced emotional disturbances (T1_HAMD‐24_: −3.00, T2_HAMD‐24_: −4.50). Moreover, improvements in emotion (ΔHAMD‐24 and ΔHAMA‐14) were significantly positively correlated with the degree of FOG‐Q improvement (ΔFOG‐Q) (r_HAMD‐24_ = 0.525, *p* < 0.05; r_HAMA‐14_ = 0.508, *p* < 0.05). Our findings are consistent with previous research. Kim et al. [33] reported that after five consecutive days of 10‐Hz rTMS applied to bilateral M1, the real stimulation group showed a mean reduction of 1.6 points in FOG‐Q scores, whereas the sham stimulation group showed a slight increase of 0.2 points. This suggests that HF‐rTMS targeting the M1 region can effectively alleviate FOG in PD patients. Yokoe et al. [24] conducted a crossover study and reported that three consecutive days of 10‐Hz rTMS applied to bilateral M1 significantly improved motor symptoms in PD patients. However, unlike our findings, their study failed to observe an improvement in emotional disturbances. This discrepancy may be attributed to differences in the baseline severity of emotional symptoms between study populations. Additionally, while our study utilized the internationally recognized HAMA and HAMD scales to assess emotional changes [38], Yokoe et al. utilized the AES, MADRS‐S, and SDS scales. Variations in scale selection may have contributed to differences in the evaluation of treatment efficacy. It is important to note that the Yokoe study applied a relatively short 3‐day stimulation protocol and did not observe any lasting therapeutic effects. In contrast, Kim et al. used a 5‐day regimen, with results showing that the treatment effects persisted for at least 1 week posttreatment. Notably, our study provided patients with a continuous 10‐day stimulation protocol. The results revealed that both FOG‐Q and UPDRS‐III scores remained lower at T2 than at T1, indicating that prolonged HF‐rTMS has a long‐term effect on both FOG and motor function, which persisted for at least 30 days. Meta‐analyses indicate that rTMS efficacy and duration depend on the total number of pulses, stimulation sessions, and intersession intervals [39]. A single rTMS session induces transient cortical excitability changes and symptom relief, typically fading within 30 min, reflecting early‐phase synaptic potentiation or inhibition. In contrast, repeated rTMS sessions accumulate cortical excitability changes in FOG patients, inducing long‐term potentiation (LTP) and facilitating a shift from transient to sustained synaptic plasticity, ensuring prolonged therapeutic effects [33].

PD‐FOG is often accompanied by cognitive decline, primarily in executive function and attention [37]. A study reported that HF‐rTMS (20 Hz) over bilateral M1 not only significantly improved motor symptoms in PD but also led to mild cognitive enhancement [40]. However, our findings showed no significant changes in MMSE or MoCA scores at any time point in either the stimulation or control group. A systematic review on the efficacy of rTMS in treating Alzheimer's disease found no difference in treatment outcomes with pulse frequencies below or at 10 Hz [41]. Most studies that demonstrated cognitive improvement employed high‐frequency pulses in the 10–20 Hz range. We speculate that the rTMS frequency in our study may not have reached the threshold needed to enhance cognitive function. Further research is needed to explore optimal stimulation parameters.

This study shows that SMA, as a combined rTMS target, can also improve FOG (T1_FOG‐Q_: −1.80, T2_FOG‐Q_: −2.20), motor function (T1_UPDRS‐III_: −8.80, T2_UPDRS‐III_: −10.65), and emotion disorders (T1_HAMD‐24_: −2.45, T2_HAMD‐24_: −2.35). Previous research supports this [42, 43], with Kim et al. [23] demonstrating that 10‐Hz rTMS on the SMA was more effective than on the motor cortex in reducing freezing episodes, highlighting SMA as a more suitable brain stimulation target. Hamada et al. [42] reported that 5‐Hz rTMS applied to the SMA once per week for 8 weeks led to an average improvement of 4.5 points in UPDRS‐III scores, significant reductions in HAMD scores, and sustained benefits for 4 weeks. A subsequent study by the same study group compared the effects of HF‐rTMS (10 Hz) and LF‐rTMS (1 Hz) on PD patients [43], revealing that while HF‐rTMS provided only transient benefits, LF‐rTMS induced more prolonged therapeutic effects. It is noted that our study confirms that HF‐rTMS applied to the SMA exerts a positive effect on motor symptoms in PD patients, with effects lasting at least 1 month. We did not, however, conduct a longer follow‐up.

Although single‐target rTMS shows efficacy in treating FOG, its therapeutic impact remains limited. Emerging research underscores the potential of multi‐target and dual‐modal NIBS strategies, such as electromagnetic or combined magnetic stimulation, in more effectively alleviating both motor and nonmotor symptoms in PD patients [44, 45]. Wang et al. [46] demonstrated that rTMS applied to M1, combined with transcutaneous magnetic spinal cord stimulation, improved gait and motor function in levodopa‐resistant PD‐FOG patients. Furthermore, Moria et al. [47] found that simultaneous tDCS targeting M1 and the left dorsolateral prefrontal cortex (lDLPFC) significantly reduced FOG severity in PD patients. Compared to single‐target stimulation, we applied bilateral M1 and SMA stimulation, leading to a more effective reduction in FOG episodes (T1_FOG‐Q_: −3.00, T2_FOG‐Q_: −4.65), improved motor function (T1_UPDRS‐III_: −10.55 T2_UPDRS‐III_: −12.30), and alleviated emotional disturbances (T1_HAMD‐24_: −4.25, T2_HAMD‐24_: −5.90). As objective measures of FOG, the TUG and SS‐180 turn test showed significantly greater improvement in the multi‐target rTMS group compared to the single‐target group (*p* < 0.001), with scores at T1 and T2 markedly lower than at T0. In contrast, the sham group exhibited no significant changes across time points. These results suggest that HF‐rTMS targeting both M1 and SMA enhances overall gait performance in PD‐FOG patients, including sit‐to‐stand transitions, straight walking, and turning. This supports our hypothesis that multi‐target rTMS is a promising therapeutic strategy for PD‐FOG. Previous studies have reported that HF‐rTMS over M1 enhances cortical excitability and increases blood oxygen level‐dependent (BOLD) signals in the SMA [48], indirectly activating its neural activity. We speculate that M1 rTMS strengthens M1‐SMA functional connectivity, facilitating synaptic activation in the SMA and restoring normal brain network dynamics to mitigate FOG and motor symptoms in PD. Moreover, the SMA is primarily involved in the preparation, planning, and initiation of movement, whereas the M1 is responsible for generating motor commands and driving muscle contractions. Therefore, applying high‐frequency stimulation to the SMA aligns more closely with the physiological direction of cortical motor information flow and may help enhance SMA‐M1 functional coordination, thereby contributing to the therapeutic efficacy of the dual‐target stimulation approach.

Previous studies have indicated that placebo responses in PD, particularly in patients with FOG, are not always prominent. For example, Lang et al. reported that placebo effects in PD are primarily mediated by endogenous dopamine release within the striatum [49]. However, this dopamine‐dependent mechanism tends to diminish in later stages of the disease or in patients with motor fluctuations. Since FOG typically emerges in the middle to late stages of PD and is often less responsive to levodopa, the patients included in our study were inherently less likely to exhibit strong placebo responses. In line with our findings, a 10‐day HF‐rTMS study by Mi et al. reported significant improvements in FOG only in the active stimulation group, with no notable changes observed in the sham group [50]. Similarly, Song et al. found that 10 Hz bilateral M1 stimulation led to marked improvements in FOG‐Q scores, timed walking tests, and emotional measures exclusively in the real stimulation group [26]. These results suggest that minimal changes in sham groups are consistent with previously reported patterns in rTMS studies targeting PD‐FOG.

Despite offering valuable insights, this study has several potential limitations. First, target localization relied on conventional methods, which could be enhanced by incorporating neuronavigation systems for greater precision. Second, PD‐FOG is classified based on dopaminergic responsiveness into three subtypes: (1) Dopamine‐responsive FOG, (2) Dopamine‐induced FOG, (3) Dopamine‐resistant FOG. Levodopa challenge tests at baseline could refine this classification, achieving personalized treatment for patients of different categories. Lastly, as “OFF”‐state patients cannot tolerate prolonged treatments, rTMS treatment and symptom assessment were conducted during the medication “ON” state, reflecting its adjunctive effects on standard pharmacotherapy and posing challenges in accurately isolating its therapeutic efficacy.

In conclusion, multi‐target stimulation of M1 and SMA outperforms single‐target approaches by effectively reducing the severity of FOG, enhancing motor function, and alleviating emotional disturbances in PD patients, with effects lasting for at least 1 month. These findings highlight its potential as a superior therapeutic for PD‐FOG.

## Conflicts of Interest

The authors affirm that there were no financial conflicts of interest associated with this study.

## Data Availability

The data that support the findings of this study are openly available in Clinical Trials Public Administration Platform at http://www.medresman.org.cn/login.aspx.
